# Identification of Gene Expression Changes Associated With Long-Term Memory of Courtship Rejection in Drosophila Males

**DOI:** 10.1534/g3.112.004119

**Published:** 2012-11-01

**Authors:** Ari Winbush, Danielle Reed, Peter L. Chang, Sergey V. Nuzhdin, Lisa C. Lyons, Michelle N. Arbeitman

**Affiliations:** * Section of Molecular and Computational Biology, Department of Biological Sciences, University of Southern California, Los Angeles, California 90089; † Department of Biomedical Sciences and Program in Neuroscience, College of Medicine, Florida State University, Tallahassee, Florida 32306; ‡ Department of Biological Science and Program in Neuroscience, Florida State University, Tallahassee, Florida, 32306

**Keywords:** long-term memory, courtship conditioning, RNA seq, courtship behavior

## Abstract

Long-term memory formation in *Drosophila melanogaster* is an important neuronal function shaping the insect’s behavioral repertoire by allowing an individual to modify behaviors on the basis of previous experiences. In conditioned courtship or courtship suppression, male flies that have been repeatedly rejected by mated females during courtship advances are less likely than naïve males to subsequently court another mated female. This long-term courtship suppression can last for several days after the initial rejection period. Although genes with known functions in many associative learning paradigms, including those that function in cyclic AMP signaling and RNA translocation, have been identified as playing critical roles in long-term conditioned courtship, it is clear that additional mechanisms also contribute. We have used RNA sequencing to identify differentially expressed genes and transcript isoforms between naïve males and males subjected to courtship-conditioning regimens that are sufficient for inducing long-term courtship suppression. Transcriptome analyses 24 hours after the training regimens revealed differentially expressed genes and transcript isoforms with predicted and known functions in nervous system development, chromatin biology, translation, cytoskeletal dynamics, and transcriptional regulation. A much larger number of differentially expressed transcript isoforms were identified, including genes previously implicated in associative memory and neuronal development, including *fruitless*, that may play functional roles in learning during courtship conditioning. Our results shed light on the complexity of the genetics that underlies this behavioral plasticity and reveal several new potential areas of inquiry for future studies.

Long-term conditioned courtship or courtship suppression in *Drosophila melanogaster* is the phenomenon whereby a male that has courted an unreceptive female for a period of time then suppresses courtship behaviors toward a subsequent female target. Although Drosophila courtship is generally considered a series of hardwired behavior (reviewed in Dauwalder 2011; Manoli *et al.* 2006), long-term courtship suppression is a complex behavioral modification reflecting a high degree of neural plasticity that is dependent upon associative memory formation (reviewed in Griffith and Ejima 2009; Kahsai and Zars 2011). During initial courtship, the male orients toward the female and taps her with his foreleg, followed by pursuit of the female and the production of wing song. Courtship continues as the male makes contact with the female genitalia using his proboscis in licking behavior. These behaviors culminate in a copulation attempt and, if the female is receptive, successful copulation (reviewed in Greenspan and Ferveur 2000; Hall 1994). Female receptivity is determined primarily by mating status. Immature virgins and recently mated females typically reject male advances by active avoidance or running away from the male, flicking their wings, kicking, and elevating their posterior abdomens, making successful copulation unlikely (reviewed in Mehren *et al.* 2004). After conditioned courtship training, the amount of time it takes for the male to engage in courtship behaviors is increased, and the number of times courtship behaviors are performed is significantly decreased.

Conditioned courtship suppression shares many of the molecular and physiologic mechanisms involved in classical olfactory conditioning, in which a fly is trained to associate a stimulus (conditioned stimulus, CS) with a noxious stimuli (unconditioned stimulus, US), such that the fly modifies its behavior when the CS is presented alone (see Busto *et al.* 2010; Davis 2005; Quinn *et al.* 1974; Siwicki and Ladewski 2003; Tully and Quinn 1985). Aversive olfactory conditioning pairs an odor as the CS with an electric shock as the US, whereas appetitive olfactory conditioning typically pairs an odor with sucrose as the US.

In the case of courtship conditioning, similar associations between the CS and US can be made because the male can differentiate the pheromone/odor profiles between immature virgins, mature virgins, and mated females (Ejima *et al.* 2005; Everaerts *et al.* 2010). It is thought that pheromones associated with mature female flies serve as the CS, whereas an aversive pheromone cue associated with mated females serves as the US (Mehren *et al.* 2004; Siegel and Hall 1979; Tompkins *et al.* 1983). Recent research suggests that the failure to successfully copulate may also act as a US for associative memory formation (Mehren *et al.* 2004), increasing the potential complexity of the memory formed. As in other learning paradigms, when a previously conditioned male encounters the CS in a subsequent trial, the male suppresses courtship in anticipation of the US.

Long-term courtship suppression is distinct from aversive and appetitive olfactory conditioning in that flies are trained to modify an ethologically relevant behavior in response to repeated and prolonged exposure to a complex stimulus, with multiple signals potentially acting as the US. Although many of the molecular mechanisms underlying long-term memory (LTM) are undoubtedly conserved between learning paradigms, such as the cAMP-PKA-CREB pathway for the induction of new gene expression in the formation of LTM, the complexity of long-term conditioned courtship suggests that unique sets of genes may also function in the formation and maintenance of courtship memory.

Furthermore, understanding the potential mechanisms involved in long-term courtship suppression would greatly increase our understanding of *in viv*o ethologically relevant memory formation. Across species and learning paradigms, increased gene transcription and protein synthesis represents a conserved mechanism necessary for LTM formation in the first few hours after training (reviewed in Roth *et al.* 2010). Later waves of transcription and translation are necessary for the persistence and extended maintenance of LTM (Bekinschtein *et al.* 2007; Katche *et al.* 2010; Miniaci *et al.* 2008). Although several key mechanisms necessary for the induction and formation of conditioned courtship memory have been identified, little is known about the transcriptional changes associated with the maintenance of long-term courtship conditioning.

In this study, we used deep transcriptome sequencing to study changes in gene transcript abundance associated with long-term courtship suppression. The use of deep sequencing methodologies allows for determination of transcriptome differences between naïve and trained flies with greater resolution and sensitivity than previous techniques (Malone and Oliver 2011), including the identification of differentially expressed genes and their isoforms. Here, we analyzed the transcriptome within head tissues from age-matched naïve and trained flies, 24 hours after a courtship-conditioning regimen that is sufficient for inducing long-term courtship suppression. Differentially expressed genes likely include those from a later wave of gene expression necessary for LTM maintenance and may differ substantially from the genes previously identified at earlier time points post-training in other learning paradigms (see Dubnau *et al.* 2003; Guan *et al.* 2011). We identified a much larger number of genes with differentially expressed transcript isoforms compared with genes with differences in overall levels of expression. Although a number of transcript isoforms are from genes previously implicated in LTM through olfactory conditioning assays, a substantial number of additional genes were identified with predicted and known functions in nervous system development, chromatin biology, translation, cytoskeletal dynamics, and transcriptional regulation. The genes identified in this study likely underlie the generation and maintenance of long-term courtship suppression that enables persistent memory for several days. The identification of additional genes that drive memory formation provide new opportunities for further study of the mechanisms behind long-term courtship suppression, some of which may be unique to this training paradigm, while others may be conserved components relevant to memory for multiple learning paradigms.

## Materials and Methods

### Drosophila strains

Flies were raised on standard cornmeal food medium at 25° on a 12-hr light and 12-hr dark cycle. We used wild-type flies of the Canton-S strain and *white* (*w*) mutant strains that have been introgressed into the Canton-S background (*w*Canton-S), previously maintained in the laboratory of Ulrike Heberlein.

### Courtship conditioning assay

**Figure 1:**
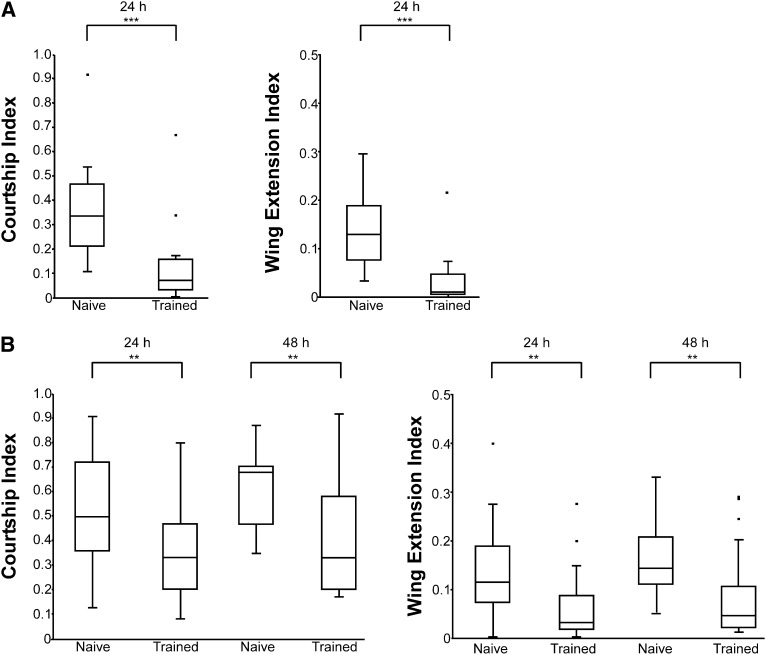
Male flies trained with mated female trainer flies show reduced courtship toward mated female tester flies compared with their naïve counterparts at both 24 and 48 hr after training. Data are presented as box plots comparing courtship and wing extension indices between naïve and trained male flies. Lower and upper box borders denote the 25th and 75th percentiles, respectively. Middle lines denote the medians. Whiskers denote the most extreme data and outliers are plotted as individual dots. (A) Subsets of naïve and trained males removed from the same group of flies on which we performed transcriptome sequencing at 24 hr after training. In this experiment, n = 16 and 17 for naïve and trained flies respectively; ***P < 0.001 in Mann-Whitney *U*-test. (B) In a separate behavioral experiment, male flies were tested at both 24 and 48 hr after training. Reduced courtship at both time points indicates long-term persistence of courtship conditioning. In this experiment, n = 20 and 21 for naïve and trained flies, respectively, at 24 hr and n = 20 for both naïve and trained flies at 48 hr; **P < 0.05 in Mann-Whitney *U*-test.

Long-term courtship suppression was induced in Canton-S male flies using methods modified from those previously described (Ishimoto *et al.* 2009; Keleman *et al.* 2007; Sakai *et al.* 2004). Pharate adult male flies were collected just before eclosion and aged individually 4 to 7 days in small cotton-plugged 5-mL tubes containing food (Figure 1A). Mated female trainers were prepared by aging *w*Canton-S virgins 2 to 6 days in groups of 20 before being mated and used as trainers. In previous studies, virgin females usually were mated by pairing groups of females with an equal number of Canton-S males for a 12- to 18-hr period before they were used as trainers (*i.e.*, Keleman *et al.* 2007). In preliminary experiments, this mating period appeared insufficient for the strains used here because a substantial portion of females remained receptive during subsequent preliminary training of naïve males. Consequently, the mating period was extended to 24 to 48 hr to establish female trainers. The majority of female trainers were actively ovulating at the end of this period ensuring that they were unreceptive during the subsequent training period.

Individual males were trained by introducing a single mated female trainer into the tube containing an individual male and pushing the cotton plug downward to create a small ~1-cm space. Training was performed overnight (12-14 hr, with light so the male flies would also learn from the visual information), during which the male periodically courts the unreceptive female, similar to spaced training protocols (McBride *et al.* 1999). We and many others have found that males do not court 100% of the time in similar assay conditions to those used here, even when the male is naïve and paired with a virgin female, which leads us to conclude that the training regimen used here is akin to spaced training. Naïve males were similarly treated, but without the introduction of a mated female. This introduces a potential confounding variable that we are unable to circumvent because the trained male performed courtship behaviors, which could also cause gene expression changes unrelated to memory formation. However, we waited 24 hours after training in an effort to allow males to recover from performing courtship behaviors. After the training period, mated females were removed, and a small subset of naïve and trained males were tested for long-term courtship suppression 24 hr later. The remaining flies were used for tissue collection (described in the section *Collection of fly head tissue and RNA extraction*). In experiments in which we tested whether memory persists to 48 hr after training (Figure 1B), male flies were collected 0-6 hr after eclosion, but were aged as described previously.

Successful induction of long-term courtship suppression was tested by comparing courtship (CI) and wing extension (WEI) indices between naïve and trained males when paired with a mated female for 10 min in a small 10-mm diameter Plexiglass chamber. Pairings were digitally recorded using Logitech Webcam Pro 2000 at two megapixel resolution and videos were analyzed using Noldus Observer XT Software Version 10.5, with the scientist blinded to the conditions (Figure 1A). Using this software, we quantified the amount of time the male fly spent performing any of the courtship behaviors. For discrete event behaviors (attempted copulation and successful copulation), we recorded behavior as a point event and coded when the behavior occurred. For nondiscrete behaviors, *i.e.*, continuous or long-lasting behaviors (pursuit of the female by following her and extension of the wing), we recorded behavior as a state event and marked when the fly initiated and ended performing the behaviors.

CI represents the ratio of time the male spends engaging in any of the courtship behaviors in the entire 10-min period. WEI represents the ratio of time the male spends extending a wing during the 10-min period and provides an additional measurement of courtship behaviors. Data are presented as box plots of the CIs and WEIs between naïve and trained males and were statistically analyzed using the nonparametric Whitney-Mann rank sum test.

### Collection of fly head tissue and RNA extraction

For each experimental condition, four biological replicates of approximately 80-100 flies were collected and treated as described previously. Naïve or trained males were snap-frozen in liquid nitrogen at the appropriate time point after training and decapitated by striking of the frozen CryoVials against a hard surface causing detachment of the head from the thorax (VWR conical bottom cryogenic vial with internal threading, 89094–802). The heads were collected on a dry ice–cooled surface and homogenized in 1 mL of Trizol (Invitrogen).

### cDNA library preparation for IIlumina sequencing

RNA purification and cDNA library preparation was as described in Chang *et al.* 2011. mRNA was purified from total RNA using the MicroPoly(A)Purist Kit (Ambion) and chemically fragmented using the Ambion Fragmentation Reagent. First-strand cDNA synthesis was performed with SuperScript Reverse Transcriptase (Invitrogen) using a combination of oligo(dT) and random hexamer primers. Second-strand synthesis was performed using DNA polymerase I in combination with ribonuclease H. cDNA fragments were blunt ended using the Epicentre End-it Repair kit and A-overhangs were generated using the Klenow Fragment (3′ to 5′ exo^-^; New England Biolabs). IIIumina sequencing adapters were ligated onto both ends of cDNA fragments using the Epicentre DNA ligation Kit. cDNA fragments were amplified by polymerase chain reaction incorporating a combination of multiplexing and indexing primers and gel-purified to isolate 200- to 550-base pair fragments. Libraries from biological replicates of each condition were sequenced on an IIIumina Genome Analyzer.

### Statistical analysis of Illumina sequencing data

Sequence reads were mapped to the Drosophila reference genome (release 5.29; 14,858 predicted genes). The reads were mapped using Tophat and analyzed for gene coverage (Trapnell *et al.* 2009, 2012). Data analysis was restricted to genes in which FPKM (fragment per kilobase of exon per million fragments mapped) values were at least 1.0 in all four biologic replicates of at least one experimental condition to ensure detected differences in transcript abundance were not due to technical differences in gene coverage for low-abundance transcripts; similar criteria were used in the large modENCODE consortium study (Roy *et al.* 2010). The algorithm Cufflinks was used to assemble mapped reads into transcripts, estimate their abundances, and test for differential expression between experimental samples (see Trapnell *et al.* 2012). For transcript isoforms, we limited our analysis to transcript isoforms with FPKM values of at least 0.5, in all four biological replicates, in at least one experimental condition.

The data were normalized using TMM normalization, implemented through the edgeR package in R (Robinson and Oshlack 2010). Statistical analysis of the data also was performed using the edgeR statistical package (Robinson *et al.* 2010), which assumes that count data are Poisson-distributed and an exact Poisson test is used for testing for significant differences between samples. q values were calculated by applying a false discovery rate adjustment to account for multiple testing (Benjamini and Hochberg 1995), which increases power but also increases the rate of type 1 statistical errors. More detail on the analysis pipeline can be found in our previous study (Chang *et al.* 2011). The Gene Ontology analysis was implemented with no test correction in the Flymine portal (Lyne *et al.* 2007).

## Results

### Trained male flies exhibit long-term courtship suppression

Spaced-training protocols result in more robust, longer-lasting memory compared with massed-training protocols, as evidenced by multiple learning paradigms across species, including *Caenorhabditis elegans*, Aplysia, Drosophila, mice, and rats (see Amano and Maruyama 2011; Beck *et al.* 2000; Carew *et al.* 1972; Kogan *et al.* 1997; Mandel *et al.* 1989; Tully *et al.* 1994). We used an overnight training protocol lasting 12−14 hr, which approximates a spaced-training protocol. During training, the male fly periodically courts the female that has mated previously (mated female) and experiences rejection behaviors. The repeated failed courtship attempts lead to suppression of courtship behaviors toward the female target by the end of the training period and when subsequently presented with a different mated female target (see Griffith and Ejima 2009).

To verify that our training regimen resulted in long-term courtship suppression, we tested LTM in a subset of naïve and trained male flies from the same group of flies on which we performed transcriptome analysis (see *Materials and Methods*). Naïve and trained flies were tested by placing individual males with a second mated female (tester female), and the courtship behaviors were quantified. An observed decrease in the amount of time spent courting (CI) or extending a wing (WEI) after training indicates courtship suppression and the formation of LTM.

As expected, trained males exhibited significantly lower levels of courtship when compared with their naïve controls (Figure 1A; *P* < 0.001 Mann–Whitney *U*-test), indicating successful induction of long-term courtship suppression. In a separate series of behavioral assays, we confirmed that long-term courtship suppression is maintained for at least 48 hr after training in our regimen. CIs and WEIs between naïve and trained flies were compared at 24 and 48 hr after training. As predicted, trained males exhibited significantly lower levels of courtship behaviors at both 24 and 48 hr after training when presented with a mated tester compared with their naïve controls (Figure 1B; *P* < 0.05 Mann–Whitney *U*-test). Other studies that used a slightly different training regimen showed that LTM persisted for at least 5 days after training (Ishimoto *et al.* 2009). Given that the memory is robust at 48 hr after training, we predict that our transcriptome analysis at 24 hr after training will identify differential gene expression associated with the maintenance and persistence of LTM.

### Transcript abundance differences between wild-type naïve and trained flies

To gain insight into the transcriptional changes underlying the persistence of long-term courtship suppression, we compared gene expression in head tissues between naïve and trained flies at 24 hr after training. Using this time point to evaluate the maintenance of LTM helps eliminate the potentially confounding effect of transcriptional changes due to courtship, mating, and social interactions that have been demonstrated in males (Ellis and Carney 2010, 2011). We analyzed whole head tissues because long-term behavioral changes may be directed by gene expression in the nervous system and/or fat body tissues (Lazareva *et al.* 2007), and dissecting these tissues leads to a stress response in gene expression, which can also confound the results. Although our approach will not identify early changes in gene expression that may be necessary for the induction or early consolidation of conditioned courtship memory, it can identify changes in gene expression that mediates the persistence and maintenance of LTM.

Illumina sequencing libraries were generated from four independent biological replicates from naïve and trained Canton-S flies. A total of 8556 (57% of all annotated genes) and 8447 (56%) genes had sufficient coverage by sequence reads in the naïve and trained males, respectively, with 8342 (56%) genes with sufficient coverage in both naïve and trained males. All subsequent analysis of differential gene expression was limited to the 8661 genes with sufficient sequence coverage in at least one experimental condition (for criteria, see *Materials and Methods*). We identified 91 genes with differential expression between naïve and trained flies 24 hr after training. Of these, 37 and 54 genes had significantly higher and lower transcript abundance, respectively, relative to naïve flies respectively (q < 0.05; supporting information, File S1).

### Analysis of transcript isoform abundance differences between naïve and trained flies

We also identified genes with transcript isoforms whose abundances differ significantly between naïve and trained male flies. A total of 11,240 isoforms from 8766 genes had sufficient coverage for further analysis (for criteria, see *Materials and Methods*). We identified 1062 isoforms with differential expression between naïve and trained flies (q < 0.05; File S2). These isoforms were the product of 787 genes and includes the 91 genes identified as having overall expression differences between trained and naïve males (see the section *Transcript abundance difference between wild-type naïve and trained flies*). This finding indicates that many of the isoforms come from genes that were not initially identified as having overall differences in expression between naïve and trained flies.

Among the 1062 transcript isoforms with significant differences in abundance, 520 and 542 had higher and lower transcript abundance, respectively, in the trained males as compared with naïve males. In many instances, different transcript isoforms encoded by a single gene were detected as having significantly higher or lower abundance in the trained males. These results demonstrate the power of using RNA-seq for detecting differences in transcript isoform abundance when applied to behavioral and learning and memory questions.

### Genes with greater abundance in trained flies

The 37 genes with significantly greater transcript abundance in trained males, as compared with naïve males, are the genes predicted to be up-regulated as a consequence courtship conditioning (see the section *Transcript abundance differences between wild-type naïve and trained flies*; q < 0.05). Using Flymine to identify overrepresented functional groups from this list (Lyne *et al.* 2007), we found that the majority of the top 15 Gene Ontology (GO; classification system for describing gene product characteristics) categories were associated with muscular cytoskeleton dynamics (File S1). Genes in this category include *Msp-300*, *actin 57B*, *sallimus*, *bent*, *paramyosin*, *myofilin*, and *CG8036*. Cytoskeletal proteins play a critical role in synaptic plasticity, cell polarity, mitosis, axon dynamics, and transport and these processes are important for the changes in neuronal architecture necessary for memory formation. However, because these genes are mainly associated with muscle structure and function, it is unknown what function they may serve in the brain, or if they are acting in muscle tissues, or other head tissues. Several of these genes have been shown to be expressed in neuronal tissues, consistent with the idea that they may be functioning to alter neuronal architecture (Chintapalli *et al.* 2007).

Two genes encoding yolk polypeptides (*Yp1* and *Yp3*) also showed greater transcript abundance in the trained males. These fat body-specific polypeptides are normally expressed in females and repressed in males as a consequence of the sex determination pathway (Belote *et al.* 1985; Burtis *et al.* 1991). However, transcriptional activation of yolk polypeptides through mechanisms outside of the sex-determination hierarchy has been reported in adults through ecdysone signaling (Bownes *et al.* 1983), and ecdysone levels are greater after courtship conditioning in trained males [(Ishimoto *et al.* 2009) see File S1 for a complete list].

### Transcript isoforms with greater abundance in trained flies

Of the 520 transcript isoforms with significantly higher abundance in trained male flies, 141 had at least a twofold difference in expression. We identified several transcript isoforms previously implicated in learning and memory (Table 1), as well as others (File S2). Among those known to function in learning and memory is *dunce* (FBtr0070522; no transcript detected in naïve males), which encodes a cAMP-specific phosphodiesterase [reviewed in (McGuire *et al.* 2005)], *fragile X mental retardation protein* (*fmr1*; FBtr0301386; fold difference: 2.99), shown previously to play a critical role in courtship conditioning (Banerjee *et al.* 2010), and the Drosophila neurotrypsin *tequila* [FBtr0076528; fold difference: 3.32 (Didelot *et al.* 2006)]. Two of these genes have additional transcript isoforms with significantly greater expression in trained flies, but these isoforms do not have twofold differences in abundance: Isoforms of *silver* (FBtr0070087; fold difference: 1.36), and *Pka-R1* (FBtr0299891; Fold difference: 1.59). Several additional genes previously associated with memory also had isoforms with significantly greater expression in trained flies. Among these were *orb2* [FBtr0076561; fold difference: 1.81 (Keleman *et al.* 2007; Majumdar *et al.* 2012)], *CaMKII* (FBtr0100146; fold difference: 1.78; reviewed in Joiner and Griffith 1997), *easily shocked* (*eas*; FBtr0074212; fold difference: 1.17; reviewed in Margulies *et al.* 2005) and *Neurexin-1* [*Nrx-1*; FBtr0301485; fold difference: 1.71 (Zeng *et al.* 2007)].

**Table 1:** Genes previously associated with learning and memory with transcript isoforms that differ significantly in abundance between naïve and trained males 24 hr after training

Flybase	Gene	Transcript	Fold Difference*^a^*	FDR q Value
FBgn0000479	*dunce*	FBtr0070522	Only in trained*^a^*	1.05E-09
FBgn0038934	*Gld2*	FBtr0084200	Only in trained*^a^*	3.19E-05
FBgn0261854	*aPKC*	FBtr0303436	3.34 up	1.63E-03
FBgn0023479	*tequila*	FBtr0076528	3.32 up	7.08E-28
FBgn0028734	*Fmr1*	FBtr0301386	2.99 up	1.07E-08
FBgn0004648	*silver*	FBtr0070085	2.79 up	1.30E-13
FBgn0003520	*staufen*	FBtr0301614	2.28 up	1.72E-09
FBgn0003396	*schnurri*	FBtr0088099	2.19 up	1.35E-09
FBgn0041111	*lilliputian*	FBtr0290041	2.10 up	8.00E-03
FBgn0035938	*orb2*	FBtr0076561	1.81 up	5.00E-04
FBgn0038975	*Nrx-1*	FBtr0301485	1.71 up	7.63E-21
FBgn0003380	*Sh*	FBtr0303902	1.64 up	2.00E-03
FBgn0000054	*Adf1*	FBtr0086113	1.64 up	1.00E-02
FBgn0259243	*Pka-R1*	FBtr0299891	1.59 up	3.30E-02
FBgn0030412	*tomosyn*	FBtr0073678	1.59 up	8.65E-07
FBgn0250753	*exba*	FBtr0078747	1.49 up	1.00E-02
FBgn0004648	*silver*	FBtr0070087	1.36 up	4.00E-03
FBgn0259246	*brp*	FBtr0299916	1.33 up	2.00E-03
FBgn0259246	*brp*	FBtr0299915	1.30 up	6.00E-03
FBgn0016917	*Stat92E*	Fbtr0100457	1.30 up	2.00E-02
FBgn0030412	*tomosyn*	FBtr0300376	1.29 up	2.00E-02
FBgn0004624	*CamKII*	FBtr0100146	1.28 up	3.00E-03
FBgn0000536	*eas*	FBtr0074212	1.17 up	2.00E-02
FBgn0086902	*kis*	FBtr0078144	1.09 up	3.00E-02
FBgn0003501	*Src64b*	FBtr0100504	11.38 down	2.59E-06
FBgn0030412	*tomosyn*	FBtr0073676	7.73 down	5.14E-14
FBgn0000536	*eas*	FBtr0074213	2.86 down	3.03E-05
FBgn0035938	*orb2*	FBtr0076564	2.65 down	2.00E-03
FBgn0004624	*CamKII*	FBtr0100148	2.31 down	2.16E-08
FBgn0038975	*Nrx-1*	FBtr0084256	2.20 down	5.80E-25
FBgn0259243	*Pka-R1*	FBtr0302639	2.07 down	2.70E-08
FBgn0000422	*Ddc*	FBtr0081166	1.89 down	1.00E-03
FBgn0000054	*Adf1*	Fbtr0086112	1.89 down	3.00E-03
FBgn0259246	*brp*	Fbtr0300542	1.65 down	6.23E-05
FBgn0035938	*orb2*	FBtr0076562	1.46 down	3.00E-02
FBgn0016917	*Stat92E*	Fbtr0089487	1.41 down	4.00E-03
FBgn0004648	*silver*	FBtr0290017	1.40 down	5.00E-03
FBgn0028734	*Fmr1*	FBtr0082198	1.40 down	1.00E-03
FBgn0003396	*schnurri*	FBtr0088101	1.39 down	3.00E-03
FBgn0003392	*Shi*	FBtr0074123	1.36 down	1.00E-03
FBgn0004648	*silver*	FBtr0070086	1.31 down	6.14E-07
FBgn0003371	*sgg*	FBtr0070467	1.28 down	2.00E-03
FBgn0037913	*fabp*	FBtr0100321	1.10 down	2.00E-03
FBgn0013334	*Sap47*	FBtr0301655	1.10 down	3.00E-02

Columns include: Flybase identification (Flybase), gene symbol (Gene), transcript identification (Transcript), fold difference, and FDR q value. FDR, false discovery rate.

a “Only in trained” indicates that these transcript isoforms had sequence reads that were only detected in trained males and not in naïve males; “up” and “down” indicate transcripts that are either up-regulated or down-regulated in trained flies, respectively.

There is increasing evidence that epigenetic modification of chromatin plays a critical role in the neuronal plasticity that underlies memory formation (reviewed in Roth *et al.* 2010). Genes encoding histone methyltransferases and histone deacetylases have been shown to be critical for Drosophila long-term courtship suppression (Fitzsimons and Scott 2011; Kramer *et al.* 2011). We identified several transcript isoforms that encode products with roles in chromatin modification that have significant and substantial greater abundance in trained males (q < 0.05 and fold difference >2). These included *su(var)3-3* (FBtr0074826; fold difference: 3.52), *E(bx)* (FBtr0072521; fold difference 2.83), *scrawny* (FBtr0077104; fold difference 2.84), *trithorax* (FBtr0082949; fold difference: 2.45), *pipsqueak* (FBtr0088277; fold difference: 2.61), and *little imaginal disks* (*lid*; FBtr0079232; fold difference: 7.60; File S2). These results further bolster the idea that chromatin modification underlies memory formation.

In addition to *dunce*, one of the transcript isoforms with the greatest fold difference is *quick-to-court* (FBtr0079014; no transcript detected in naïve males), whose molecular function is unknown but has been shown to play a critical role in regulating male courtship behavior, with males harboring mutations in this gene courting more quickly than wild-type males (Gaines *et al.* 2000). Consistently, here it appears that greater expression of *quick-to-court* is correlated with reduced levels of courtship behaviors in trained males.

We determined which tissues in the adult head had significantly high expression for the genes with up-regulated transcript isoform expression after courtship training, as determined by the previous Flyatlas study (Chintapalli *et al.* 2007), and assessed here using the Flymine portal (Lyne *et al.* 2007). Comparison with the Flyatlas study found that of the 520 transcript isoforms with significantly greater abundance in trained male flies 274, 247, and 246 of the genes that encode these isoforms had significantly up-regulated expression in the adult brain, eye and head, respectively; the Flyatlas study did not examine other cell types of the adult head. Taken together with the GO analysis (see File S2), these results suggest that we identified genes that function in the nervous system, as well as in other adult head tissues.

### Genes with reduced transcript abundance in trained flies

Genes with significantly lower transcript abundance in trained male flies (q < 0.05), as compared with naïve male flies are those predicted to be repressed in response to courtship conditioning. The majority of overrepresented GO categories in this group were associated with metabolic processes related to stress and immunity (File S2); this finding is consistent with other studies demonstrating reduced expression of genes that underlie the stress response and immune activity in sexually active males (Carney 2007; McKean and Nunney 2001). Ribosomal proteins and genes with products that function in protein translation were also among the overrepresented GO categories. Genes include: *RpL15*, *RpL38*, *RpS28b*, *RpL41*, *RpS25*, *RpS29*, and *RpL39*. Flies bearing a *P* element mutation in the ribosomal protein encoding gene, *RpL19*, showed defects in LTM formation in the context of aversive olfactory training (Akalal *et al.* 2011). These previous studies together with our data may suggest a role for down-regulation of protein synthesis as a consequence of courtship conditioning at this time point.

Among genes with large differential abundance (fold-difference ≥2) were *dArt 4*, which encodes a histone arginine methyltransferase and may act as a transcriptional cofactor (Cakouros *et al.* 2004; Urwyler *et al.* 2007), and *piefke*, which encodes a protein that contains a Pipsqueak DNA binding domain and may play a role in neurogenesis and chromatin remodeling (Schwendemann and Lehmann 2002). A gene encoding a member of the Dynein complex, *short wing*, also had robust differential expression and plays a critical role in intracellular transport, cell polarity, and dendritic branching (Zheng *et al.* 2008). Given the known developmental role of these genes, it is possible that these genes are playing a role in modifying the neuronal architecture during adult stages, similar to their earlier developmental roles. In addition, two neuropeptide precursor encoding genes, *nplp2* and *nplp3*, also had lower abundance in trained flies. There is increasing evidence for a role for neuropeptides in Drosophila learning and memory, olfactory behaviors, and courtship (reviewed in Nassel and Winther 2010). It will be interesting to determine these genes normal roles in regulating courtship behaviors and how their expression is modified in the context of courtship suppression.

### Isoforms with lower transcript abundance in trained flies

Among the 542 transcript isoforms with lower abundance in the trained condition, 146 had a twofold or greater difference in expression (File S2). Interestingly, isoforms from several genes previously found to be required for learning and memory (summarized in Table 1) had substantially lower transcript isoform abundance including (fold difference >2): *CaMKII* (FBtr0100148; fold difference: 2.31), *orb2* (FBtr0076564; fold difference: 2.63), *eas* (FBtr0074213; fold difference: 2.86), *Nrx-1* (FBtr0084256; fold difference: 2.20), and S*rc64B* (FBtr0100504; fold difference: 11.38). An additional isoform of *orb2* (FBtr0076562; fold difference; 1.46) showed reduced abundance, along with two additional isoforms of *silver* (FBtr0070086; fold difference: 1.31 and FBtr0290017; fold difference 1.40), an additional isoform of *fmr1* (FBtr0082198; fold difference 1.40) and *schnurri* (FBtr0088101; fold difference 1.39). These results point to the complexity of understanding the formation of LTM, with our identification of genes previously shown to be required for LTM in loss-of-function and null mutants having isoforms that appear to be repressed in these trained flies at this time point. Our study also highlights the differences in molecular processes between the induction of memory formation and the maintenance of memory and suggests that understanding the role of individual transcript isoform over a period of time during memory formation will be important.

The gene *pipsqueak* that encodes a product that has a role in epigenetics also had an isoform with reduced transcript abundance in trained flies (FBtr0088278; fold difference: 2.53). Pipsqueak proteins are known to have developmental roles (Grillo *et al.* 2011) and *pipsqueak* mutants exhibit defects in olfaction (Sambandan *et al.* 2006). As such, *pipsqueak* may play a role in long-term courtship suppression by altering the processing of olfactory information. As is the case with other genes, *pipsqueak* also had an isoform that had higher abundance in trained flies (see above).

### Differentially expressed isoforms with known roles in nervous system development

Long-term courtship suppression likely involves changes in neuronal connectivity, synaptic growth, synapse formation, and synaptic organization, which would be mediated in part by genes with roles in neuronal development. We identified genes with known roles in nervous system development with transcript isoforms that are significantly higher and lower in abundance in trained males (File S1 and File S2). Interestingly, the list of transcript isoforms that have lower abundance in trained males included the gene *fruitless* (FBtr0083646; fold difference 3.80; non-sex-specific transcript), which is necessary for the potential for male courtship behaviors but had not been previously implicated in long-term courtship suppression (reviewed in Dauwalder 2011; Manoli *et al.* 2006). This result supports the idea that *fruitless* possesses a role in adult neuronal plasticity, in addition to its known developmental role, which although suggested previously (Belote and Baker 1987), has also been questioned (reviewed in Yamamoto *et al.* 1997).

## Discussion

Understanding learning and the formation of complex memory requires not only the identification of mechanisms necessary for the induction and formation of memory but also the identification of mechanisms involved in the maintenance of LTM. The ethological relevance, complexity, and persistence of long-term courtship suppression in Drosophila make this behavior an ideal model for studying the molecular mechanisms involved in the maintenance of LTM. Using RNA sequencing and genome-wide transcriptome analysis, we analyzed gene expression in naïve flies and conditioned flies 24 hr after training to identify differential gene expression associated with the maintenance of long-term courtship suppression. In the behavioral experiments, courtship suppression toward mated females was observed at both 24 and 48 hr after training. Because the head tissues were collected 24 hr after training, we hypothesize that many of the genes for which we detected differential gene expression contribute to lasting changes in neural circuitry that underlies the persistence of long-term courtship suppression beyond 24 hr.

Drosophila male courtship behavior and the underlying neural circuitry are complex because males must process a variety of olfactory and visual sensory inputs while also performing the courtship dance. Although the neural circuitry underlying these behaviors is genetically established by adulthood (reviewed in Greenspan and Ferveur 2000), the fact that learning can occur, suggests it remains plastic and responsive to life experiences. We identified a set of genes with differential expression 24 hr after training that were previously associated with the formation of long-term conditioned courtship memory or in other forms of associative memory (Table 1). Whereas previous studies showed these genes were necessary for the induction and formation of memory, our results showing differential regulation 24 hr after training suggest additional roles for these genes in memory maintenance.

Long-term maintenance of memory may also require the increased expression of a set of genes that is not necessary for the acquisition of memory at earlier time points. For example, the Aplysia cytoplasmic polyadenylation element binding protein (CPEB) is necessary for the maintenance of LTM at 48 and 72 hr after training but not for acquisition of memory at 24 hr (reviewed in Darnell and Richter 2012). Interestingly, recent research reported a similar role for the Drosophila Orb2A isoform in the maintenance of long-term courtship memory (Majumdar *et al.* 2012). We found that *Orb2*, a Drosophila homolog of *CPEB*, was significantly more abundant in trained males 24 hr after training, further suggesting a conserved role of this gene family for the maintenance of LTM across species. This result also points to the evolutionary conservation of the molecular mechanisms in memory formation across highly divergent species, with very different cognitive abilities.

Given that memory can last several days to years, depending on the species examined, several mechanisms that are not fleeting must contribute to sustained differences in neural activity. Sustained differential regulation of gene expression could contribute to LTM and is postulated to occur in part through chromatin modifications, with increasing evidence for a role of epigenetic regulation underlying memory formation (reviewed in Roth *et al.* 2010; Roth and Sweatt 2009). Our results provide further evidence that chromatin modification is important in the context of courtship conditioning. Given all the tools available for molecular-genetic studies in Drosophila, it will be possible to determine specific changes in chromatin modification, within the neurons known to be functionally important for courtship conditioning memory formation and retrieval.

Some genes may contribute to long-term courtship suppression by directly acting within the neurons that underlie male courtship behaviors, including genes that earlier in development specified the fate of these circuits. Cellular development and the long-term storage of memory share many similarities, as recognized through studies in Aplysia and mammals (reviewed in Day and Sweatt 2011). We found several genes with known roles in neuronal development, axon guidance, and synaptic growth were differentially expressed after courtship conditioning (File S1 and File S2). The observation that *fruitless*, a gene that specifies the fate of the neurons that underlie male courtship, had lower transcript abundance in trained males, suggests it has a critical role in adults that will be interesting to investigate further. In another study it was shown that a male could remember the mating status of the female with whom he trained, which was reflected in his behaviors, only showing reduced courtship with females of the mating status with which he trained (Ejima *et al.* 2005). Therefore, long-term courtship suppression is not simply reflecting an overall reduced activity of the circuits that underlie courtship but a much more complicated interplay between sensory functions and higher-order brain processing centers acting on/with the activity of neurons that mediate courtship.

This study provides insight into gene expression changes that may function in the maintenance of LTM. Future studies analyzing additional time points after training, in defined subsets of neurons, and within other learning paradigms will build a picture of the waves of gene transcription associated with the formation and maintenance of LTM and also will reveal some of the complex interplay among neurons. These genome-wide analyses will continue to highlight phylogenetically conserved molecular mechanisms necessary for learning and memory. An important future step will be to determine behaviorally the contributions of individual genes and transcript isoforms to the different aspects of memory formation, maintenance, and retrieval in a cell-specific manner. *Drosophila*, with its powerful molecular-genetic tools, will continue to provide a highly valuable behavioral model for understanding memory formation.

## Supplementary Material

Supporting Information
