# Supplementary material for: Identification of Gene Expression Changes Associated With Long-Term Memory of Courtship Rejection in Drosophila Males
Source: G3 (Bethesda). 2012 Nov 1;2(11):1437–45. doi: 10.1534/g3.112.004119 (PMC3484674; doi:10.1534/g3.112.004119)
Supplement: Supporting Information [file supp_2_11_1437__index.html]

Supporting Information 

# Identification of Gene Expression Changes Associated With Long-Term Memory of Courtship Rejection in Drosophila Males

## Supporting Information for Winbush *et al.*, 2012

**Files in this Data Supplement:**

- File S1 - .xlsx, 47 KB
- File S2 - .xlsx, 387 KB
